# Perforated Gangrenous Gallbladder in an Asymptomatic Patient

**DOI:** 10.7759/cureus.7728

**Published:** 2020-04-18

**Authors:** Mehdi Faraji, Rachel Sharp, Edgar Gutierrez, Kiran Malikayil, Ali Sangi

**Affiliations:** 1 Radiology, Louisiana State University Health Sciences Center, Shreveport, USA; 2 Family Medicine, Louisiana State University Health Sciences Center, Shreveport, USA; 3 Radiology, Brookwood Baptist Medical Center, Birmingham, USA

**Keywords:** perforated, gangrenous, cholecystitis, asymptomatic

## Abstract

Acute cholecystitis or inflammation of the gallbladder is a common cause of hospitalizations. A percentage of those patients will progress to gangrenous cholecystitis and perforation. This medical emergency can lead to peritonitis, which has increased morbidity and mortality. The first-line modality for the diagnosis of acute cholecystitis is an ultrasound, but if it is inconclusive, then a computed tomography (CT) scan may be beneficial. Gangrenous cholecystitis and perforation have been reported in asymptomatic diabetic patients secondary to diabetic neuropathy and/or gallbladder ischemia leading to nerve denervation. Yet, here we present the case of an asymptomatic non-diabetic patient with gangrenous gallbladder perforation that was treated with antibiotics and drain placements. Diagnosis and treatment involve the collaboration between primary care, interventional, and diagnostic services to appropriately manage these patients. This case demonstrates that clinicians should have a low threshold to conduct CT scan of the abdomen, especially when there is a sudden resolution of pain.

## Introduction

Gangrenous cholecystitis (GC) has been described since 1894, and figures vary depending on the percentage of acute cholecystitis (AC) that GC makes up. GC patients tend to be sicker than the average AC patients, and their surgical procedure tends to be more challenging and is associated with an increased risk of morbidity and mortality when compared to all causes of AC [1]. GC risk factors include diabetes mellitus, males, older age, elevated white blood cells, and coronary heart disease [2,3]. GC is the progression of AC, which is commonly due to obstruction of the cystic duct due to an impacted stone, which commonly dislodges on its own and does not progress to GC. If the stone does not dislodge, then there will be vascular compromise and epithelial injury leading to ischemia locally and necrosis of that region’s gallbladder wall. These patients will require early interventions to decrease morbidity and mortality [2,3]. Ultrasound is the first-line imaging modality, and if it is inconclusive, then a computed tomography (CT) scan can assist. On ultrasound, thickening of the gallbladder wall is expected, but one study showed that 28% of patients do not have any finding on ultrasound. On CT scan, GC will show gas in the wall or lumen of the gallbladder, discontinuous gallbladder wall, and/or pericholecystic fluid. Untreated GC can lead to abscess formation and/or peritonitis [4]. There have been reports of loss of abdominal pain post-perforation due to possible nerve denervation, especially in diabetic patients with neuropathy, but here we present the case of a patient who did not have diabetes and had no pain but was found to have a perforated gangrenous gallbladder [5].

## Case presentation

**Figure 1:**
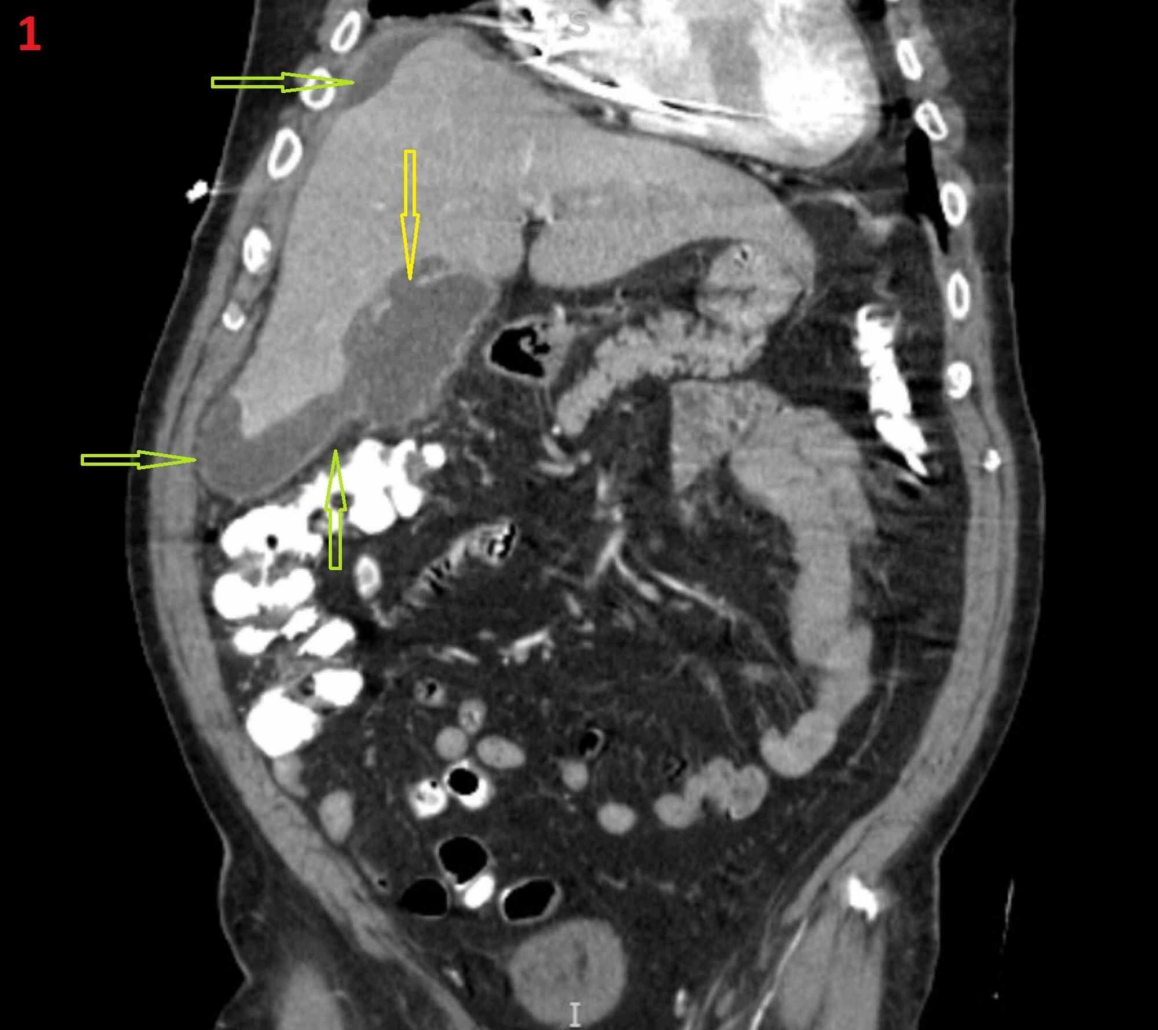
Coronal abdomen and pelvis computed tomography (CT) scan. Perihepatic fluid collection (green arrows) with hepatomegaly. There is discontinuity of the gallbladder wall (yellow arrow).

A 71-year-old patient with a history of hypertension, coronary artery disease, myocardial infarction, coronary artery bypass graft, atrial fibrillation, and pacemaker placement presented with mild right upper quadrant abdominal pain to the emergency department. He described the pain as sharp and non-radiating. He stated that he had a decrease in appetite in the last several days, but he denied nausea, vomiting, and diarrhea. His vital signs were all stable. He had not consumed alcohol or smoked in five years. His medications included warfarin 9 mg daily, atorvastatin 40 mg daily, carvedilol 12.5 mg twice daily, and hydrochlorothiazide 25 daily. His physical examination was positive for mild tenderness in the right upper quadrant of his abdomen without rebound. His labs were normal, including his white blood cells, liver function test, and coagulation profile. His HIV test, hepatitis B core IgM, hepatitis B surface antigen, and hepatitis C antibody were negative. His chest and abdominal X-ray were normal, and abdominal ultrasound was also normal and only showed hepatomegaly. He continued to improve symptomatically and was pain-free; therefore, we had planned to discharge him, but being cautious, we did a CT scan and saw that the patient had discontinuous gallbladder mucosa and intramural and pericholecystic fluid pockets indicative of GC (Figure 1).

**Figure 2:**
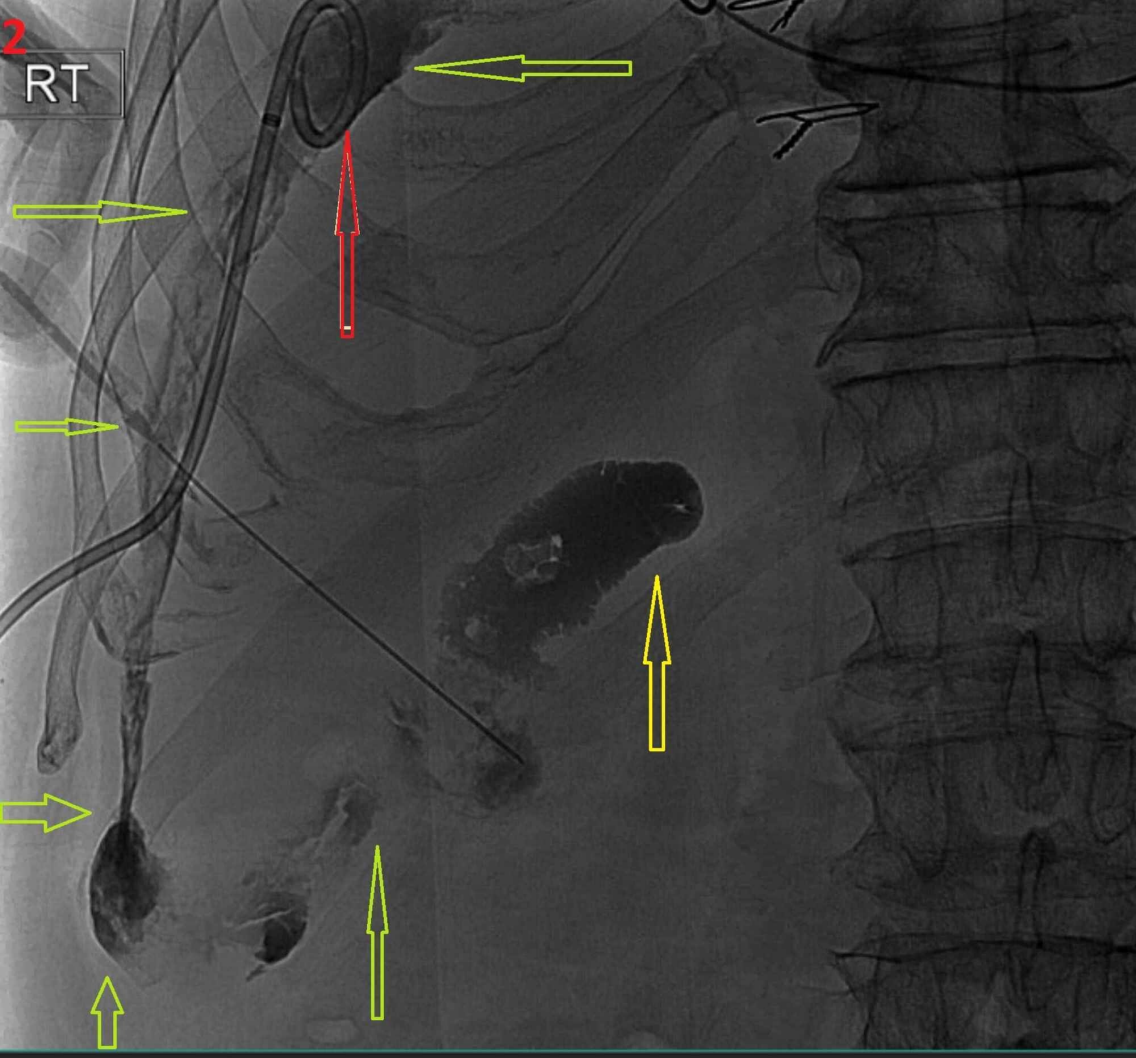
Fluoroscopy of the gallbladder and perihepatic space. Diluted contrast showed partial filling of the gallbladder (yellow arrow) and communication into the perihepatic space (green arrows). The drain was placed in the perihepatic space (red arrow).

An interventional radiologist placed one drain with 400 mL of brownish fluid removed from the perihepatic region (Figure 2).

**Figure 3:**
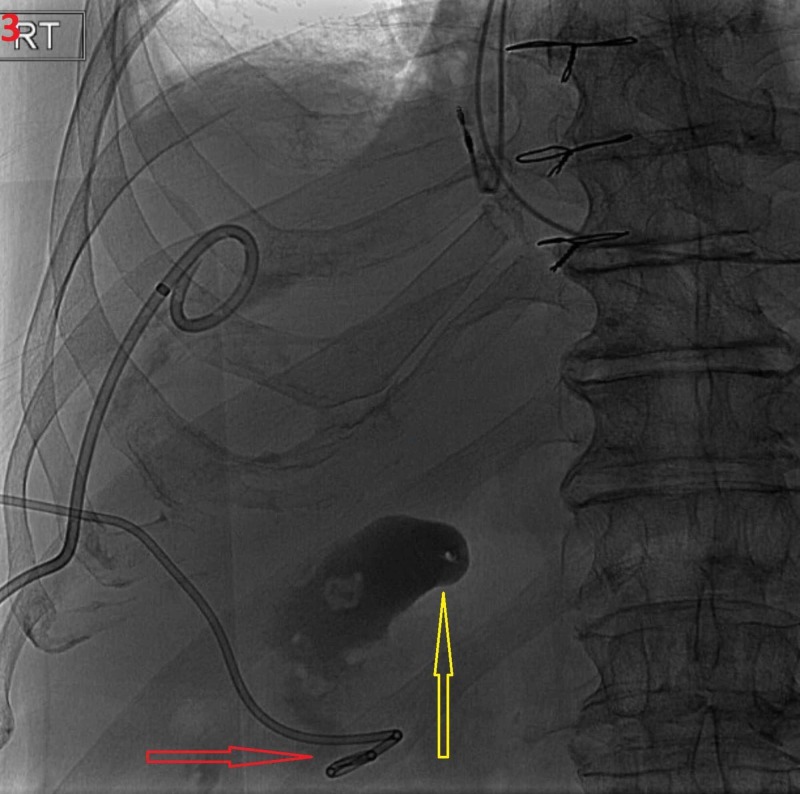
Fluoroscopy of the perforated gallbladder. The second drain (red arrow) was placed into the perforated gallbladder (yellow arrow).

Another drain was placed into the gallbladder under fluoroscopy as contrast showed rupture of the gallbladder and communication with the perihepatic space (Figure 3).

Samples were sent for culture showing Klebsiella pneumonia and vancomycin-resistant Enterococcus, with treatment with linezolid for six weeks. The patient was followed up with resolution of pain and removal of drains in one month.

## Discussion

The patient presented with certain features of AC, including right upper quadrant abdominal pain, with resolution of his pain. Due to our caution, we performed abdominal CT imaging and found the patient to have perforated GC. This led the interventional radiologist to place two drains in the patient to clear the bilious fluid, which has high morbidity and mortality from possible abscess and/or peritonitis. This case shows that the medical community as a whole should have a low threshold for CT imaging in a patient who had resolution of his right upper quadrant abdominal pain and negative ultrasound when the gallbladder was thought to be involved.

AC can progress to GC and lead to perforation, and this perforation can lead to bilious fluid around the liver [1]. The liver has a capsule that is composed of two layers: Glisson’s capsule (thick fibrous internal layer) and the outer visceral peritoneum derived serous layer. The subcapsular space is the space between the Glisson’s capsule and the liver itself. The gallbladder fundus is attached to the liver and covered by the same serosal layer as the liver, and thus bile from perforated gallbladder will expand into the potential space known as the subcapsular space [6]. There is an increased risk of GC from AC if the patient has diabetes, coronary artery disease, and elevated white blood cells, and/or is older [2]. There have been reports of asymptomatic patients with perforated gallbladder due to diabetic neuropathy or possible nerve denervation in ischemic perforated gallbladder wall but, rarely, any report of a perforated gangrenous gallbladder in a non-diabetic patient. This report describes this very unusual presentation with a favorable outcome.

## Conclusions

Perforation is the natural progression of GC that progresses from AC if not found early and requires immediate intervention to decrease morbidity and mortality. Here we present an unusual case where the patient was asymptomatic and ready for discharge, but due to a cautious clinician, the patient was found to have a perforated GC on CT imaging. This led to the placement of two drains to decrease the risk of peritonitis and/or abscess formation and its associated increased morbidity and mortality. Thus, the appropriate management requires a collaborative effort among primary care, interventional, and diagnostic services, with a low threshold for CT imaging.
